# Selective attention to pain and empathy: Studying frequent blood donors

**DOI:** 10.1002/brb3.2841

**Published:** 2022-12-01

**Authors:** Sina Omyan, Mahdi Mazidi, Ali Khatibi

**Affiliations:** ^1^ Institute for Cognitive Science Studies Tehran Iran; ^2^ Centre for the Advancement of Research on Emotion, School of Psychological Science The University of Western Australia Perth Australia; ^3^ Centre of Precision Rehabilitation for Spinal Pain University of Birmingham Birmingham UK; ^4^ Centre for Human Brain Health University of Birmingham Birmingham UK

**Keywords:** altruistic behaviour, empathy, pain, selective attention

## Abstract

**Introduction:**

Empathy is an interpersonal experience that enables understanding of other's emotions and can lead to altruistic behavior such as blood donation. Cognitive theories of empathy refer to selective attention as one of its cognitive dimensions. The current study examined if individuals who engage in altruistic behavior are characterized by a distinct pattern of selective attention to observation of pain in others.

**Methods:**

We recruited 50 volunteer blood donors. Half (*n* = 25) of the volunteers donated for a self‐declared altruistic reason, and the other half of the volunteers donated blood for a health‐related reason. We assessed the individuals’ self‐reported empathy with the Interpersonal Reactivity Index (IRI). We then measured the individuals’ selective attention toward faces expressing pain in a pictorial dot‐probe task.

**Results:**

Consistent with the proposed hypothesis, participants who donated blood out of altruism reported significantly higher empathic concern on the IRI than those who donated blood for a health‐related reason. The altruistic donors also showed significantly greater selective attention toward facial expressions of pain. Moreover, among all donors, self‐report empathic concern on the IRI was significantly correlated with greater selective attention toward faces expressing pain.

**Discussion:**

These findings suggest that altruistic individuals not only show higher levels of empathy, but also attend more to the pain of others. Limitations, implications, and suggestions for future research are discussed.

## Graphical abstract

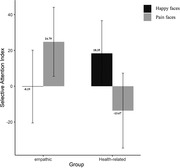

## 1 INTRODUCTION

Empathy is an interpersonal experience that enables emotion understanding and facilitates social interactions (Decety & Jackson, 2004). Empathy implicates cognitive processes (e.g., interpretation, attention) and affective responses (e.g., compassion, distress), and can lead to altruistic behaviors such as blood donation (Finck et al., 2016). Most research on empathy for pain has shown that pain observation triggers affective distress (e.g., discomfort), vicarious pain (both painful and non‐painful vicarious sensations), and empathic responses (e.g., feelings of compassion and tendency to help) for others’ pain (Giummarra et al., 2015; Khatibi et al., 2022). Facial expressions are specifically evolved for social communication and provide a key channel for conveying emotions (Horstmann, 2003). In the context of pain, facial expressions are salient social signals of need for help or potential physical threat (Khatibi & Mazidi, 2019; Williams, 2002), which can also elicit empathic responses in the observer (Leslie et al., 2004) and encourage altruistic behavior from them to alleviate the pain (Preston & Waal, 2002).

To date, only a few studies examined the association between selective attention and empathy in the context of pain. Bi et al. (2021) compared those with high and low levels of self‐reported empathy in their attention to pain‐related images using a dot‐probe paradigm. The pain‐related and neutral stimuli were images of foot, forearm, or hand in painful or nonpainful situations, respectively. They found that the high empathic participants showed significantly greater attention to pain‐related stimuli compared to participants with lower levels of empathy. In another study, Pilch et al. (2020) examined the role of empathy on selective attention to pain faces by manipulating participants’ perspective taking as an essential component of empathy. Participants were presented with pain and neutral face pair and were informed that expressions were of people who had taken part in a painful task.

They instructed two groups of participants to either take self‐perspective (imagining how they would feel about what is happening) or other perspectives (imagining how the person is feeling) while viewing face pairs, while the third group received no instruction. Significant greater attention to pain faces was found among the groups who were asked to engage in perspective‐taking compared to the control group.

While attention is considered critical in empathy and altruism, it remains to be assessed how the current findings would translate into actual behavioral responses. This question could be addressed by examining patterns of selective attention to pain‐related information among individuals who actively engage in an altruistic action to help others. Recruiting this group would allow to test if the patterns of attentional allocation among individuals who engage in prosocial behaviors out of empathy are significantly different from the control group and to examine if this difference is characterized by greater attention to pain‐related information among those with higher levels of empathy. Therefore, the current study recruited a group of volunteer blood donors, who gave blood out of empathy at least two times per year. Volunteer blood donors are individuals who donate blood, plasma, or cellular components with their own free will and receive no monetary reward (Dhingra, 2012). Because the procedure of blood donation is associated with pain (France et al., 2013), to control for it, we also recruited a control group from individuals who donated blood to improve their own health.

The aim of the current study was to examine the association between altruistic behavior and selective attention to pain‐related information. We hypothesized that the empathic blood donors would show greater attention to pain faces compared to those who donate blood to improve their health. If found to be supported, it would provide further evidence that selective attention to pain in others implicates higher levels of empathy. Alternatively, if this is the case that selective attention to pain in others does not play a role in empathy, no differential patterns of attention to pain between groups and no association between selective attention and empathy would be found.

## 2 METHODS

### 2.1 Participants and procedure

Blood donors who visited a blood donation center in Ankara were approached by the experimenter before the donation. The power calculation was performed using Gpower (Faul et al., 2007) to ensure there is sufficient power to detect the intended selective attention difference between the groups. A sample of 24 participants (minimum 18 years old) for each group was determined adequate for finding the selective attention effect with a medium effect size (*d* = 0.6) and a statistical power of 0.8 for α = .05 (Bi et al., 2021). Participants had normal vision (or corrected‐to‐normal). They were able to speak and understand Turkish easily. The other eligibility criterion was making a blood donation at least three times in the last 24 months. None of the participants had psychiatric or neurological disorders (self‐reported). APA's ethical practice guidelines have been followed in the planning, conducting, and reporting the findings of this study. The ethics committee of the department of psychology at Bilkent University approved the study. All the participants signed written consent prior to their participation. The blood donation procedure took 45 min on average.

A total of 50 participants were recruited and were asked for their reason for visiting the blood donation center. Among them, 25 individuals donated blood for empathic reasons (they had specific blood type, or they had read that the center is low in the reserve), and 25 individuals believed that blood donation is good for their health and were advised by their doctor to donate blood. Data from five participants were excluded from analyses due to technical problems in recording the responses. The final sample included 23 empathic blood donors and 22 health‐related blood donors.

### 2.2 Self‐report measures

#### 2.2.1 The interpersonal reactivity index (Davis, 1983)

The Interpersonal Reactivity Index (IRI) is a 28‐item scale assessing empathy. It comprises four subscales: empathic concern, perspective taking, fantasy, and personal distress. Empathic concern represents the feeling of compassion and concern toward others. Perspective‐taking assesses the ability of the individual to adopt others’ point of view. Fantasy shows the tendency to transpose oneself into fictional situations and characters, and personal distress measures feelings of anxiety in tense interpersonal situations. Each item is rated on a 5‐point Likert scale range (0 = Does not describe me well; 4 = Describes me well). The range of subscale is 0–28. Good reliability, construct validity, and internal consistency have been reported for IRI (Davis, 1983; De Corte et al., 2007). Internal consistency for the IRI (Cronbach's alpha) in the present study's sample was .77.

#### 2.2.2 Pain catastrophizing scale (Sullivan et al., 1996)

The Pain Catastrophizing Scale (PCS) is a self‐report measure consisting of 13 items and three subscales: rumination, magnification, and helplessness that assess catastrophizing thoughts and feelings related to painful experiences. Responses are rated on a 5‐point Likert scale, from 0 (not at all) to 4 (always). The higher the total score, the more the patient exhibits pain catastrophizing. The PCS has been shown to be a reliable and valid measure for both samples with and without psychopathology (Khatibi et al., 2014; Ranjbar et al., 2020). Internal consistency for the total score (Cronbach's alpha) in the present study's sample was .88.

### 2.3 Dot probe

Selective attention was assessed using a pictorial version of the dot‐probe paradigm (MacLeod et al., 1986). This task has been shown to be sensitive to individual differences in selective attention to pain‐related information (Mazidi, Vig et al., 2019; Mohammadi et al., 2012). The task was programmed using Affect 4 that is a free software package for implementing psychophysiological and psychological experiments and is commonly used for tasks that require registering response latencies (Spruyt et al., 2010). Stimuli were presented on a 15.6 in. laptop monitor and consisted of photos of 10 adults (5 females) that were taken from the Montreal Database of Facial Expressions (Roy et al., 2007). This is a standardized and one of the most commonly used database in pain studies. Roy et al. (2007) created their database that includes basic emotions as well as pain and neutral expressions using experienced actors as their models, and then empirically validated the stimuli through participant’ rating of the presented emotions. Each trial started with a fixation point (+) at the center of the screen for 500 ms. After the fixation point was removed, a pair of faces appeared on the screen, one above the other. One of the faces was always emotional (either pain or happy expressions), and the other face was neutral from the same model. The faces remained for 500 ms, and then a probe (*) was presented in the same spatial location of either the emotional or neutral face. Participants were instructed to respond as quickly as they could without compromising accuracy. They were required to press the ¯ key if the probe appeared at the previous location of the bottom face or the ­ arrow key in case the probe appeared in the previous location of the top face. The trial ended when participants pressed one of the keys or after 2000 ms. The ITI was between 800 and 1200 ms. The task was delivered in two separate blocks and started with 16 practice trials with neutral images from different landscapes. Experimental trials consisted of 40 trials with painful/neutral face pairs and 40 trials with happy/neutral face pairs. All experimental trials were randomly presented in the same block. The presentation of faces was randomized (displayed four times in every possible combination). Participants completed the task before the questionnaire, and the average completion time of the task was 6 min.

### 2.4 Data preparation

Trials with incorrect responses were removed (less than 1% of trials). No significant differences were detected between two groups in the frequency of erroneous responses [*t*(42.71) = 0.53, *p* = .59]. The dot‐probe task's outlier responses were dealt with using the Winsorizing method recommended by Price et al. (2015). This is a method that minimizes missing values and maximizes the power by reallocating outliers to the closest value in the non‐outlier (valid) distribution. Employing the recommended values, RTs higher or lower than 1.5 interquartile range between the 25th and 75th percentiles of each participant's distribution were reassigned to the nearest valid score (Price et al., 2015).

Selective attention index was calculated using the following formula (Khatibi et al., 2009):

$$\mathrm{Selective}\;\mathrm{attention}\;\mathrm{index}:\;\;\;\left(\mathrm{tupl}-\mathrm{tlpl}\right)+\left(\mathrm{tlpu}-\mathrm{tupu}\right)/2$$

where t is the target face, p is the dot probe, u is the upper position, and l is the lower position.

According to the above formula, a positive selective attention value reflects greater attention toward the corresponding emotional facial expression, while a negative selective attention value signifies attentional avoidance from the corresponding emotional facial expression. Separate selective attention indices were calculated for trials with happy faces and those with pain faces.

### 2.5 Data analysis

Independent *t*‐tests were used to test group differences on questionnaire measures and demographic information. Two‐way ANOVAs were conducted with group (empathic donors vs health‐related donors) as the between‐group factor and target stimuli type (painful vs happy) as the within‐group factor, with selective attention index as the dependent variable. The α was set to .05 for analyses. Significant effects were followed up using *t*‐tests to clarify differences, and Cohen's *d* and partial eta‐squared were used to compute effect sizes. Correlations between attentional indices and self‐report measures were also examined using Pearson correlation coefficient. To control the false discovery rate, Benjamini–Hochberg correction was employed, which has been shown as a more powerful procedure than comparable methods for controlling the traditional family‐wise error rate (Benjamini & Yekutieli, 2001).

## 3 RESULTS

### 3.1 Preliminary analyses

No significant differences were found between empathic donors and health‐related donors in terms of age or fantasy (Table 1). The empathic donor group showed significantly greater empathy, perspective‐taking, personal distress, and rumination and magnification subscales of PCS (see Table 1). Differences in empathy‐related variables were expected to be related to the groups’ status (i.e., their main reason for blood donation). Therefore, we did not see the need to control for these variables. We repeated our analyses with PCS rumination and magnification subscales as covariates. Because the Analysis of Covariance (ANCOVA) and the ANOVA yielded similar pattern of results, only the latter are reported here^1^

**TABLE 1:** Comparison between characteristics of two groups, mean (SD)

	Empathic donors	Health‐related donors	*t*(df)	*p*‐Value
Age	34.30 (9.86)	32.36 (8.97)	0.69 (43)	.494
IRI empathic concern	14.91 (6.84)	7.73 (4.62)	4.14 (38.75)	< .001
Fantasy scale	15.69 (5.49)	12.73 (4.44)	1.98 (43)	.053
Personal Distress	14.35 (7.11)	7.86 (5.64)	3.38 (43)	.002
Perspective‐taking	14.91 (5.17)	10.50 (4.98)	2.91 (43)	.006
PCS (total score)	22.48 (7.03)	16.82 (7.00)	2.70 (43)	.005
PCS—rumination	9.48 (5.14)	4.91 (4.07)	3.29 (43)	.002
PCS—magnification	7.04 (2.23)	5.59 (2.50)	2.05 (43)	.046
PCS—helplessness	5.95 (2.91)	6.32 (3.68)	−0.36 (43)	.716

Abbreviations: IRI, interpersonal reactivity index; PCS, pain catastrophizing scale.

### 3.2 Selective attention analysis

**FIGURE 1:**
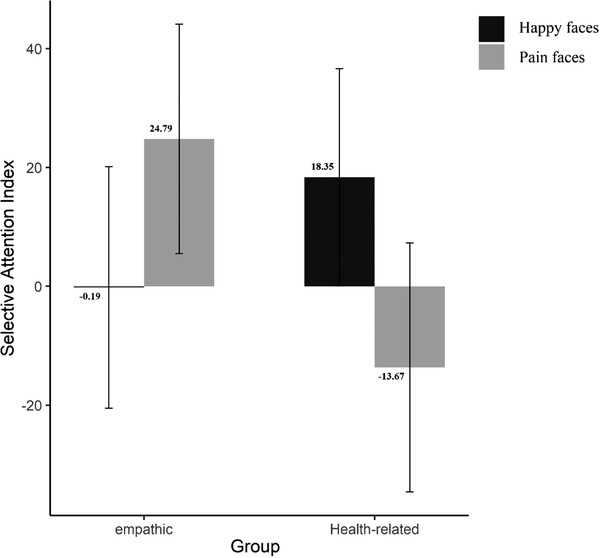
Selective attention index for pain and happy faces for two groups (errors bars represent 95% confidence interval, and numbers above or below the bars show the exact mean of selective attention index for each condition and group)

A 2 (groups: empathic donors, health‐related donors) × 2 (stimuli type: pain, happy) ANOVA was performed to find out whether those who donated blood out of empathy and those who donate for health‐related reasons differed in selective attention to painful faces. There was no main effect of group [*F*(1, 43) = 0.98, *p* = .33, *η_p_^2^* = 0.022] or type of stimuli [*F*(1, 43) = 0.15, *p* = .69, *η_p_^2^* = 0.004]; however, the interaction effect for group and type of stimuli was significant [*F*(1, 43) = 10.17, *p* = .003, *η_p_^2^* = 0.19]. Independent *t*‐tests indicated a significant difference between participant groups for pain expressions [*t*(43) = 2.81, *p* = .007, *d* = 0.85] as empathic donors displayed greater attention toward faces expressing pain, while health‐related donors displayed greater attention to happy faces (see Figure 1). No significant difference was found between groups in their selective attention index to happy faces [*t*(43) = −1.40, *p* = .16].

### 3.3 Correlations and regression analyses

Among all participants, greater selective attention to pain faces was significantly associated with higher IRI empathic concern (*r* = .72, *p* < .001), greater personal distress (*r* = .63, *p* < .001), and greater rumination subscale of PCS (*r* = .65, *p* < .001). No more significant correlations were found between selective attention index to pain or happy faces and other variables (all *rs* < .29, all *ps* > .054). To further examine the association between empathy, catastrophizing, and selective attention to pain, we ran two regression models in which IRI and PCS scores were entered as predictors and selective attention index for pain and happy faces as outcome variables. For the model with selective attention index to pain faces as the outcome variable, only the empathic concern subscale of the IRI was a significant positive predictor (*b* = 3.79, *t* = 2.89, *p* = .006). None of the predictors was significant for selective attention index to happy faces (*bs* < 4.06, *t*s < 1.69, *p* > .100).

## 4 DISCUSSION

The aim of our study was to test the putative link between altruism and greater selective attention toward pain. We demonstrated that participants who donate blood for an altruistic cause were characterized by higher levels of empathy and greater attention toward pain faces compared to participants who donate blood to improve their own health. This finding suggests that selective attention toward pain faces might be a marker of higher levels of empathy and might motivate individuals to involve in altruistic behavior. A potential reason for this might be that greater selective attention to painful facial expression relies more on affective than cognitive component of empathy (Grynberg & Maurage, 2014). These results suggests that the tendency to be engaged in prosocial behavior is linked to a higher level of empathy and, consequently, higher attentional allocation to others in pain. Therefore, people with higher levels of empathy may be motivated by other‐oriented and care‐based responses by demonstrating selective attention toward others’ pain. This is in line with the notion that higher empathy may facilitate attentional processing of information that signals others’ needs in order to perceive those needs (Wu et al., 2017).

However, not all previous studies found that higher empathy is associated with greater attention to pain‐related information. For example, Hong et al. (2020) presented participants with pain‐related and neutral stimuli that consisted of images of back and face regions with and without cupping marks on the skin, respectively, while assessing their attention by an eye‐tracker. They found a significant negative association between empathy and selective attention to pain‐related images. The different findings of Hong et al. (2020) might be due to the different stimuli type they employed. Specifically, their stimuli did not show that the models were experiencing pain due to cupping as all used faces were showing neutral expressions.

The findings of the current study were in line with the findings of Bi et al. (2021) and Pilch et al. (2020). Specifically, similar to Bi et al. (2021) who found significant positive association between empathy and selective attention to pain‐related information (i.e., images others’ different body parts in painful situations), significant correlation was found between higher empathy and greater selective attention to pain‐related information (pain faces) in the current study. Moreover, in line with Pilch et al. (2020), the current study provided further support that higher‐order cognitive factors may affect early attentional processes that are typically assumed to be automatic and stimulus‐driven. Specifically, the empathic concern subscale of the IRI was the significant positive predictor of greater attention to pain faces. The empathic concern assesses feelings of sympathy and concern when observing others are in unfortunate situations (Davis, 1983). Pilch et al. (2020) has shown that participants who took a self‐perspective view of other's pain (i.e., to imagine the pain they saw as their own) demonstrated greater attention to pain faces compared to observers who were not instructed to do so, which is close to empathic concern.

There are some strengths and weaknesses associated with the current study. To the best of our knowledge, the current study was the first study that examined patterns of selective attention among individuals who actively engage in blood donation out of empathy and compared it with the well‐matched control group of participants who also donated blood but due to other reasons. This strength noteworthy, the limitations of this study should also be noted and acknowledged. First, it must be recognized that causal inferences cannot be made because of the cross‐sectional nature of this study. While the pattern of findings is consistent with the possibility of the causal associations between empathy and selective attention to pain in others, testing this causal possibility will require further research. Future studies designed to test the causal accounts between empathy and selective attention can adopt longitudinal designs or manipulate attentional patterns or empathy to examine potential causal relationships between them (Hayes & Rockwood, 2017). For example, selective attention to pain‐related stimuli could be manipulated using the attention bias modification procedures to examine if a successful change of selective attention could affect participants’ empathy toward others’ pain and their tendency to help them (Khatibi & Mazidi, 2019). Second, the study would have benefited from the inclusion of other negative facial expressions as stimuli (e.g., angry or sad faces). These additions would have permitted us to examine the specificity of the observed selective attention to pain‐related stimuli compared to other negative emotions. Future studies can address this limitation by including stimuli of other negative faces. Third, the current study assessed selective attention employing the dot‐probe task that relies on RT data to infer the allocation of attention to different stimuli and provides only a snapshot of attentional allocation (Cisler & Koster, 2010). Future researchers can overcome this limitation by employing eye‐tracking methodology. Eye‐tracking method can assess attention continuously and as a dynamic phenomenon, which provides the researcher with a more precise assessment of the time‐course of attention to different stimuli (Armstrong & Olatunji, 2012). Eye‐tracking also permits the presentation of more complex and ecologically valid stimuli instead of only two images/words that is used in the dot‐probe paradigm (Soleymani et al., 2022). Other future directions include examining the potential moderating role of attentional control in the association between empathy and selective attention to pain (Mazidi, Dehghani et al., 2019; Ranjbar et al., 2020), employing eye‐tracking methodology to examine attentional allocation to pain information in a continuous manner and test its association with empathy. Fourth, different components of selective attention, that is, facilitated attentional engagement and difficulty disengagement, can differentially contribute to empathy. The current study was not designed to distinguish between these different components; however, future studies can address this possibility by adapting and employing the available paradigms that permit the assessment of different components of selective attention (Clarke et al., 2013; Rudaizky et al., 2014). Finally, a critical avenue for future research in this area is to examine the potential interactive role of empathy and selective attention to pain‐related information on both the relationship between caregivers and patients with pain as well as caregivers’ mental health and burnout. There are some pieces of preliminary evidence that caregivers’ selective attention to pain is significantly and positively associated with reporting pain behaviors in patients; however, the role of empathy has not been examined in this association (Mohammadi et al., 2015). It remains a question for future studies if the function of empathy and selective attention or the relationship between these constructs is the same for caregivers of pain patients and people from community that has been recruited for previous studies in this filed.

In conclusion, the current study provides evidence for the association between empathy and selective attention to pain‐related information in others and implies that selective attention to others’ pain can be implicated in higher empathy and the tendency to engage in prosocial behavior. This finding has implications for the cognitive theories of empathy and encourages further investigation into the complex associations between selective attention, empathy, and prosocial behavior.

## CONFLICT OF INTEREST

The authors declare that there is no conflict of interest regarding the current submission.

### PEER REVIEW

The peer review history for this article is available at https://publons.com/publon/10.1002/brb3.2841

## Data Availability

The data that support the findings of this study are available on request from the corresponding author. The data are not publicly available due to privacy or ethical restrictions.

## NOTE

1 A participant had the overall mean reaction time above 3 SD of the sample; removing that participant did not change the output of any of the analyses reported, therefore, we chose to keep that participant.
